# Comparison of the gut microbiota of college students with the nine balanced and unbalanced traditional Chinese medicine constitutions and its potential application in fecal microbiota transplantation

**DOI:** 10.3389/frmbi.2023.1292273

**Published:** 2023-12-20

**Authors:** Qinhong Huang, Lihui Yang, Guannan Cai, Yongdie Huang, Shian Zhang, Zhenwei Ye, Jing Yang, Chuhui Gao, Jiaxuan Lai, Lyu Lin, Jihui Wang, Ting Liu

**Affiliations:** ^1^ Innovation Centre for Advanced Interdisciplinary Medicine, Key Laboratory of Biological Targeting Diagnosis, Therapy and Rehabilitation of Guangdong Higher Education Institutes, The Fifth Affiliated Hospital of Guangzhou Medical University, Guangzhou, China; ^2^ Department of Neurosurgery, Zhujiang Hospital, Southern Medical University, Guangzhou, China; ^3^ The First Clinical School of Guangzhou Medical University, Guangzhou, China; ^4^ The First Affiliated Hospital of Jinan University, Guangzhou, China; ^5^ The First Affiliated Hospital of Shantou University Medical College, Shantou, China

**Keywords:** gut microbiota, traditional chinese medicine constitution, FMT, high-fat (HF) diet, tradition al chinese medicine

## Abstract

Fecal microbiota transplantation (FMT) has been tested for the prevention and treatment of various intestinal and extra-intestinal diseases, but its efficacy is not stable, which may be due to the lack of an optimized method for screening high-quality donors. The low efficiency and high cost of donor screening are also obstacles to the clinical application of FMT. In this study, we tested the efficiency of the constitution theory of traditional Chinese medicine (TCM) in screening high-quality FMT donors. College student volunteers were sorted into either the balanced TCM constitution (BC) or unbalanced TCM constitution (UBC) groups, with the latter group comprising eight different constitution types, and the gut microbiota profiles of each UBC were compared with that of BC. Subsequently, the success rates of the qualified donors of BC and UBC volunteers were compared. Finally, the anti-obesity effect of FMT, obtained using the fecal microbiota of BC and UBC donors, was tested on mice with high fat diet-induced obesity. The results showed that the gut microbiota of BC and UBC volunteers were significantly different. There was a higher proportion of qualified FMT donors in the BC volunteer group than in the UBC volunteer group. Moreover, the experiment in mice showed that the fecal microbiota of BC and UBC volunteers conferred different anti-obesity effects. Overall, TCM constitution could be a reference for FMT practice. Our study presents a new idea, namely, using TCM constitution theory to efficiently screen high-quality FMT donors.

## 1 Introduction

The gut microbiota is closely related to health and diseases (Kataoka, 2016; Hills et al., 2019). However, the composition of the gut microbiota varies greatly among individuals, making it difficult to define the profiles of abnormal and normal healthy gut microbiota (Huttenhower et al., 2012; Vujkovic-Cvijin et al., 2020). Having a clear standard for a healthy gut microbiota is of exceeding importance, since it not only provides a direction for improving gut microbiota composition but also provides a clear target for the selection of fecal microbiota transplantation (FMT) donors. FMT, as the most rapid, direct, and effective means of recovering gut microbiota dysbiosis, has been clinically tested around the world for a variety of diseases, including both intestinal and parenteral diseases (Collado et al., 2015). For intestinal diseases, its cure rate for pseudomembranous enteritis caused by *Clostridium difficile* infection is as high as 90%, but is approximately only 40% for inflammatory bowel disease (Lai et al., 2019). For parenteral diseases and conditions, such as obesity, diabetes, metabolic syndrome, autism, and depression, the cure rate is much lower and varies greatly across the reported small-scale clinical trials (Falony et al., 2019; Napolitano and Covasa, 2020; Yu et al., 2020; Borkent et al., 2022). The donor has been considered to be the key factor in such cases. Therefore, it is expected that a standard reference for a healthy gut microbiota may help to improve FMT efficacy by preventing the screening of donors without a reference criterion.

However, there is no specific criterion for identifying the healthiest gut microbiota. The comparison between people with or without a specific disease provides only some information about the characteristics of the unhealthy gut microbiota. In addition, the profiles of the healthiest gut microbiota among clinically healthy people are not well established. The super-donor phenomenon occurred occasionally in the practice of FMT, in which the fecal microbiota of the donor was quite effective in all FMT regardless of indications and recipients (Wilson et al., 2019). Therefore, super donors may provide a reference of the healthiest gut microbiota. However, super donors are so rare that there were not enough data to compare and summarize the gut microbiota characteristics of different super donors. To create a general reference of a healthier gut microbiota, we determined that identifying the healthiest people among overall healthy people may help in achieving this goal.

The body constitution theory of traditional Chinese medicine (TCM) provided us with a clue as to how to distinguish the healthiest people. According to the theory, there are nine constitution types: the balanced constitution (BC) and eight unbalanced constitutions (UBCs); namely, the qi-deficiency constitution (QDC); the yang-deficiency constitution (YADC); the yin-deficiency constitution (YIDC); the phlegm–dampness constitution (PDC); the dampness–heat constitution (DHC); the blood stasis constitution (BSC); the qi-stagnation constitution (QSC); and the inherited special constitution (ISC) (Qi, 2005). People with unbalanced constitutions usually do not have a simple single constitution, but a complicated constitution entailing two or more UBCs. It has been proven that a specific UBC is linked to the susceptibility to developing specific diseases (Wang, 2012; Liang et al., 2020). People with a balanced constitution are regarded as the healthiest and least likely to develop diseases (Wang, 2012). The practice of TCM in this context involves taking measures that regulate UBCs toward a BC; this is the core idea of disease prevention and therapy (Li et al., 2019). Therefore, people with a balanced constitution may provide a reference of the healthiest gut microbiota.

The fact that the gut microbiota profiles of people with a BC and those with UBCs are different has been shown in a few studies. The gut microbiota structure and metabolic characteristics of people with YIDC, YADC, and PDC have been reported and compared with those of people with a BC separately by several studies (Yingshuai et al., 2015; Ruo-xi et al., 2018; Xue et al., 2020). Nevertheless, the data from a few studies were not enough to fully define the gut microbiota profile of a BC through comparison with UBCs, and the first reason for this was their small sample size. Second, the previous studies may have neglected some important factors such as the age and health status of the test participants, which are also variables related to the gut microbiota, since detailed participant information for such variables was not provided. More importantly, most people have a complicated TCM constitution rather than a simple single constitution. The previous studies also did not identify any of the test participants’ constitutions. In all, a single TCM constitution, as the single gut microbiota variable, may not have been controlled well enough in the previous studies, which may disrupt understanding of the true features of the gut microbiota profiles of a BC and of each UBC.

In the present study, we recruited healthy college students within a very narrow age range (i.e., 20 ± 2 years) as our study participants, who were of a similar health status, had similar dietary habits, and led a relatively healthy lifestyle. Student volunteers were first screened by a psychological health professional, and the responses to a dietary and lifestyle questionnaire were used to exclude participants with poor psychological and physical health, who had an abnormal diet and led an unhealthy lifestyle, who were alcohol drinkers, and who were smokers. Subsequently, the TCM constitutions of the healthy volunteers were identified using the questionnaire developed by the TCM scientist Wang. The proportions of the qualified FMT donors with a BC and with UBCs, and the effects of FMT on mice with obesity induced by a high-fat diet, were compared using the fecal microbiota of BC and UBC donors to check the potential application of the TCM constitution theory in FMT.

## 2 Materials and methods

### 2.1 Volunteer recruitment and TCM constitution identification

The study has been approved by the institutional ethics review board of the Fifth Affiliated Hospital of Guangzhou Medical University (ref. KY02-2022-08-02). Lectures and pamphlets about the gut microbiota, FMT, and the idea of the present study were given to college students to attract their interest. Subsequently, questionnaires that covered participants’ psychological health, dietary habits, and lifestyle were sent out and taken back when fully filled. The students with poor physical and psychological health, who had an unhealthy diet and led an unhealthy lifestyle, who were alcohol drinkers, and who were smokers were excluded from the study.

The remaining healthy students went on to identify their TCM constitutions by using the questionnaire developed by the famous TCM scientist Wang (Wang et al., 2006). In brief, there are nine types of TCM body constitutions: BC, QDC, YADC, YIDC, PDC, DHC, BSC, QSC, and the ISC. The last eight are collectively named unbalanced constitutions (UBCs). The questionnaire consists of seven or eight questions for each TCM constitution, and the questions were carefully chosen to cover the main traits of each constitution. The participants answered questions by choosing “never”, “rarely”, “sometimes”, “often”, and “always” according to their actual feeling about their own body condition. A score of between 1 and 5 was assigned to each choice. An algorithm was then used to transform the scores to a 100-point system (**Supplementary Table 1**). Every participant got nine scores, ranging from 0 to 100, for each of the nine constitutions.

For the criterion of BC, when the BC score was ≥ 60 and those of all eight UBCs were < 30, the participant was defined as having a BC. When the BC score was ≥ 60, those of all eight UBCs were < 40, and that of one or more was ≥ 30, the participant was defined as being BC inclined. All other participants were regarded as having a non-BC constitution. For the criterion of UBCs, taking the QDC as an example, when the QDC score was ≥ 40, the participant was defined as having a QDC, when the QDC score was between < 40 and ≥ 30, the participant was defined as being QDC inclined, and when the QDC score was ≤ 30, the participant was regarded as having a non-QDC constitution (**Supplementary Table 1**). The identification of the other seven UBCs followed the same way. When a participant was defined as having one specific constitution and none of the other eight constitutions, we regarded he/she as having a simple single constitution, and the participant was selected for further study. Informed consent to participate in the research study was then obtained and fecal samples were taken from those participants.

### 2.2 Analysis of gut microbiota using 16S rRNA gene sequencing

Fecal samples were collected from participants, who were defined as having a simple single constitution, and frozen at −80°C for further tests. Until the end of the research project, or after enough samples were collected, the samples were subjected to gut microbiota analysis. Total DNA was extracted using a stool DNA extraction kit, following the procedures of the kit manual. The DNA integrity was checked using an ultraviolet imager after the processes of agarose gel electrophoresis and ethidium bromide staining. Subsequently, DNA purity and concentration were measured using an ultraviolet spectrophotometer. The same amount DNA from each sample was taken for the PCR amplification of the V3 + V4 region of 16S rRNA gene with barcode-labeled primers 341F and 806R. The sequencing library was then constructed using an Illumina library building kit. The DNA fragments of the library were checked using an Agilent 2100 Bioanalyzer, and the quantification of the library was performed using Qubit™ 3.0 and q-PCR. Last, the sequencing of the qualified library was performed using the Illumina HiSeq platform. The original sequencing data were analyzed according to a standard biological information process, which entailed read assembly, tag filtration, and chimera removal. The SILVA database (version 132) was used for taxonomic annotation. The ordinary analysis for the gut microbiota included β-diversity analysis, which shows the differences among groups, and the analysis of α-diversity metrics, including microbial richness, evenness, and diversity. Most importantly, the relative abundance of the gut microbiota at the phylum, class, order, family, genus, and species levels was analyzed. All the sequencing data have been submitted to SRA (Sequence Read Archive), a publicly available repository of the National Center for Biotechnology Information (NCBI), and can be accessed via the following link: https://www.ncbi.nlm.nih.gov/search/all/?term=PRJNA876832.

### 2.3 FMT donor screening from college volunteers with simple single TCM constitutions

The FMT donor screening was according to the Chinese expert’s consensus on standardized methodology and the clinical application of FMT (2022) (Qiyi and Huanlong, 2022). The screening examination projects included the following: (1) hematology screening, a routine blood test; a liver and kidney function test; the measurement of electrolyte and C-reactive protein levels; and tests to detect the hepatitis virus (A, B, C, D, and E), human immunodeficiency virus (HIV), syphilis, Epstein–Barr virus (EB virus), cytomegalovirus, Nematoda, and amebic protozoa. (2) Stool screening: fecal occult blood test, and screening for *Clostridium difficile, Campylobacter, Salmonella, Shigella*, Shiga toxin-producing *Escherichia coli*, parasite eggs, vesicles, parasites, spores, norovirus, carbapenem-resistant *Enterobacteriaceae* (CRE), extended-spectrum β-lactamases (ESBL), and methicillin-resistant *Staphylococcus aureus* (MRSA). All the above indices were examined or detected following the normal procedures of a clinical laboratory in our hospital, and when all the index values were within the normal reference range, the volunteer was considered a qualified FMT donor.

### 2.4 Fecal microbiota transplantation from human donors to mice with obesity

Specific pathogen-free (SPF) C57B/L6 mice (male, aged 6–8 weeks) were purchased from the Guangdong Medical Laboratory Animal Center. The animals were raised in the Laboratory Animal Center of Guangzhou Medical University in an SPF environment, that is, one with a temperature of 22°C and a 12 h light/12 h dark cycle. All mice experiments were approved by the Laboratory Animal Ethics Committee of Guangzhou Medical University (KY01-2020-03-11). The mice were fed with either a normal diet (ND; with a fat-to-energy ratio of 10%) or a high-fat diet (HFD; with a fat-to-energy ratio of 60%) for 6 weeks. The feed was purchased from the Guangdong Medical Laboratory Animal Center (ND, catalog number: D12450B; HFD, catalog number: D12492). From the third week of HFD, all mice were intragastrically administered with a mixed solution of antibiotics (ampicillin, 25 g/L; vancomycin, 25 g/L; neomycin, 25 g/L; metronidazole, 25 g/L; and gentamycin sulfate, 25 g/L) (10 μL/g, 250 mg/kg body weight) once per day for 7 days to eliminate their original gut microbiota. Subsequently, a total of 25 mice were randomly divided into five groups, on average, according to their designated treatments: (1) ND group; (2) HFD group; (3) HFD + FMT BC donor group (with a normal weight, and balanced constitution); (4) HFD + FMT YIDC donor group (a skinny individual with YIDC); and (5) HFD + FMT PDC donor group (a obese individual with PDC).

The fresh stool of qualified FMT donors with a simple single constitution (BC, YIDC, and PDC) was taken, weighed, and mixed with five times volume of sterile saline containing 15% glycerin. Subsequently, the mixture was homogenized and filtered through a 70-µm filter, and the filtrate was packed in a 2-mL centrifuge tube and frozen at −80°C. Before FMT, the fecal solution was taken out and thawed in a 37°C water bath, and then centrifuged at 2,000 g for 15 min. The supernatant was removed and the solid crude fecal microbiota at the bottom was weighed, and suspended in sterile saline at a final concentration of 0.3 g/mL. From the fourth week of HFD and the end of the antibiotic treatment, the mice were intragastrically administered with the crude fecal microbiota solution (10 μL/g body weight) every 2 days for 6 weeks.

The body weights and food and water intake of the mice were recorded weekly. Three days prior to the end of experiments, the glucose tolerance of the mice was measured using a glucometer and blood glucose test card (Accu-Chek® Active test strips; Roche Diagnostics GmbH, Mannheim German) following the card instruction manuals: after fasting for 10 hours, mice were orally gavaged with 1M/L glucose (10 μL/g body weight). Blood was taken from the tail tips at 0 min, 15 min, 30 min, 60 min, and 120 min, and the blood glucose content was measured simultaneously when the blood was taken. At the end of the experiments, the mice were killed under an anesthesia state. Their subcutaneous fat and epididymis fat mass were separated and weighted.

### 2.5 Statistics

The data were presented as means ± SEMs. The statistical significance of the differences were evaluated using one-way or two-way analysis of variance, followed by Tukey’s *post hoc* multiple comparison test. The data with a superscript symbol (*) were significantly different (*p < 0.05; **p < 0.01; and ***p < 0.001). Except for the 16S rRNA gene sequencing analysis, all analyses were performed using GraphPad Prism version 9.00 for Windows (GraphPad Software Inc., CA, USA).

## 3 Results

### 3.1 TCM body constitutions of college students

A total of 1,218 college students (622 females and 596 males) completed all the questionnaires, including the TCM constitution questionnaires and health and lifestyle questionnaires. The ages and body mass indexes (BMIs) of the participants were within a very narrow range (20 ± 2 years and 21.02 ± 4.37 kg/m^2^, respectively). The participants also led similar lifestyles and had a similar health status (questionnaire data not shown). In general, for every individual, his/her TCM body constitution could take one of the following forms: (1) one or more specific UBCs, plus one or more specific UBC inclined (**Table 1**); (2) BC inclined, plus one or more specific UBCs inclined (**Table 2**); or (3) simple single BC or UBC (**Table 3**). In fact, most individuals have complicated TCM constitutions, that is, one of the first two forms. As shown in the tables, only 18.7% of students had a balanced constitution (BC), which is considered to be the healthiest constitution and is associated with the lowest risk of developing diseases, whereas the rest of the students (76.0%) had unbalanced constitutions (UBCs) (**Tables 1**–**3**), which are considered to be clinically healthy in youth but more disease prone in later life, according to the TCM theory. The population distribution of the nine types of constitutions was not even. Among the eight UBCs, most individuals had QDC, YADC, YIDC, and DHC, followed by PDC, BSC, and QSC, only a few had ISC (**Tables 1**–**3**). There was also a gender-based differentiation; there were more BC males than BC females, and for some specific UBCs, such as BSC and QSC, there were more girls (**Tables 1**–**3**). For the single simple constitution, only a few individuals had a specific single constitution, and most of them had a BC instead of UBCs (**Table 3**).

**Table 1:** Sum of TCM constitutions of college students with one or more specific UBCs plus one or more specific UBC inclined.

	Total		Male		Female	
**Number**	1218		596		622	
**Age, years**	20±2		20±2		20±2	
**BMI, kg/m^2^**	21.02±4.37		21.76		20.32	
**BC n, (%)**	218	17.90%	111	18.62%	107	17.20%
**QDC n, (%)**	288	23.65%	131	21.98%	157	25.24%
**YADC n, (%)**	230	18.88%	104	17.45%	126	20.26%
**YIDC n, (%)**	247	20.28%	129	21.64%	118	18.97%
**PDC n, (%)**	200	16.42%	97	16.28%	103	16.56%
**DHC n, (%)**	205	16.83%	94	15.77%	111	17.85%
**BSC n, (%)**	220	18.06%	82	13.76%	138	22.19%
**QSC n, (%)**	196	16.09%	78	13.09%	118	18.97%
**ISC n, (%)**	148	12.15%	83	13.93%	65	10.45%

BMI, body mass index; BC, balanced constitution; QDC, qi-deficiency constitution; YADC, yang-deficiency constitution; YIDC, yin-deficiency constitution; PDC, phlegm-dampness constitution; DHC, dampness-heat constitution; BSC, blood stasis constitution; QSC, qi stagnation constitution; ISC, inherited special constitution.

**Table 2:** Sum of TCM constitutions of college students with BC inclined plus one or more specific UBCs inclined.

	Total		Male		Female	
**Number**	1218		596		622	
**Age, years**	20±2		20±2		20±2	
**BMI, kg/m^2^**	21.02±4.37		21.76		20.32	
**BC n, (%)**	218	17.90%	111	18.62%	107	17.20%
**QDC n, (%)**	288	23.65%	131	21.98%	157	25.24%
**YADC n, (%)**	230	18.88%	104	17.45%	126	20.26%
**YIDC n, (%)**	247	20.28%	129	21.64%	118	18.97%
**PDC n, (%)**	200	16.42%	97	16.28%	103	16.56%
**DHC n, (%)**	205	16.83%	94	15.77%	111	17.85%
**BSC n, (%)**	220	18.06%	82	13.76%	138	22.19%
**QSC n, (%)**	196	16.09%	78	13.09%	118	18.97%
**ISC n, (%)**	148	12.15%	83	13.93%	65	10.45%

BMI, body mass index; BC, balanced constitution; QDC, qi-deficiency constitution; YADC, yang-deficiency constitution; YIDC, yin-deficiency constitution; PDC, phlegm-dampness constitution; DHC, dampness-heat constitution; BSC, blood stasis constitution; QSC, qi stagnation constitution; ISC, inherited special constitution.

**Table 3:** Sum of TCM constitutions of college students with simple single BC or UBC.

	Total		Male		Female	
**Number**	1218		596		622	
**Age, years**	20±2		20±2		20±2	
**BMI, kg/m^2^**	21.02±4.37		21.76		20.32	
**BC n, (%)**	228	18.72%	128	21.48%	100	16.08%
**QDC n, (%)**	16	1.31%	13	2.18%	3	0.48%
**YADC n, (%)**	11	0.90%	2	0.34%	9	1.45%
**YIDC n, (%)**	11	0.90%	3	0.50%	8	1.29%
**PDC n, (%)**	3	0.25%	2	0.34%	1	0.16%
**DHC n, (%)**	15	1.23%	10	1.68%	5	0.80%
**BSC n, (%)**	2	0.16%	0	0.00%	2	0.32%
**QSC n, (%)**	5	0.41%	4	0.67%	1	0.16%
**ISC n, (%)**	1	0.08%	0	0.00%	1	0.16%

BMI, body mass index; BC, balanced constitution; QDC, qi-deficiency constitution; YADC, yang-deficiency constitution; YIDC, yin-deficiency constitution; PDC, phlegm-dampness constitution; DHC, dampness-heat constitution; BSC, blood stasis constitution; QSC, qi stagnation constitution; ISC, inherited special constitution.

### 3.2 Gut microbiota profiles of BC compared with UBC volunteers

**Figure 1:**
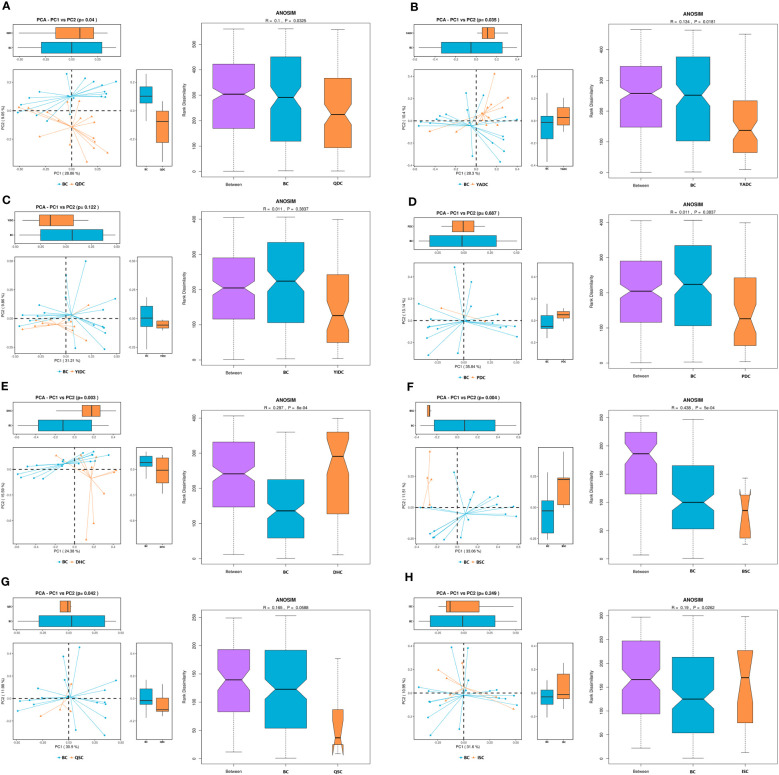
The β-diversity of BC gut microbiota compared with UBCs. **(A)** Principal component analysis (PCoA) and analysis of similarities (ANOSIM) of BC and QDC gut microbiota. **(B)** PCoA and ANOSIM of BC and YADC gut microbiota. **(C)** PCoA and ANOSIM of BC and YIDC gut microbiota. **(D)** PCoA and ANOSIM of BC and PDC gut microbiota. **(E)** PCoA and ANOSIM of BC and DHC gut microbiota. **(F)** PCoA and ANOSIM of BC and BSC gut microbiota. **(G)** PCoA and ANOSIM of BC and QSC gut microbiota. **(H)** PCoA and ANOSIM of BC and ISC gut microbiota. The PCoA scatter plot shows differences in the gut microbiota between the BC and UBC groups. Each point represents a sample, and the different colors represent the different experimental groups. The closer the points are, the more similar the samples are. ANOSIM is another index to show the gut microbiota difference between groups. When the rank between groups is higher than that of other groups, it indicates that the difference between groups is greater than that within groups. *R* ∈ (−1, 1), *R* > 0, indicates a significant difference between groups, and *R* < 0 indicates that intragroup differences are greater than intergroup differences. If the grooves of the two groups do not overlap, it shows that their medians have significant differences. The reliability of statistical analysis is expressed as *p*, and a *p*-value < 0.05 indicates significant differences. (BC, balanced constitution, *n* = 18; QDC, qi-deficiency constitution, *n* = 16; YADC, yang-deficiency constitution, *n* = 13; YIDC, yin-deficiency constitution, *n* = 11; PDC, phlegm–dampness constitution, *n* = 2; dampness–heat constitution, DHC, *n* = 11; BSC, blood stasis constitution, *n* = 5; QSC, qi-stagnation constitution, *n* = 5; and ISC, inherited special constitution, *n* = 7).

The people who have a BC are considered to be the healthiest according to the TCM theory. By comparing the gut microbiota profiles of BC and UBC people, we expected to be able to describe the healthiest gut microbiota among generally healthy people. Therefore, we collected the fecal samples of the participants who were identified as having a simple single constitution. The β-diversity results showed that the gut microbiota profiles of people who had the same TCM constitution shared more similarity, and were obviously different to those of people who had different TCM constitutions (**Supplementary Figures 1A, B**). The difference between the gut microbiota profile of the BC and each of the eight UBC gut microbiota profiles was significant, as indicated by principal component analysis (PCoA) plots and an analysis of similarities (ANOSIM) index (**Figures 1A–H**). The β-diversity results showed that richness of the BC gut microbiota was generally lower than that of the UBCs, except for QDC and BSC, as indicated by the observed species, and the Chao1 and ACE index values (**Supplementary Figures 2A–H**). The diversity and evenness of the BC gut microbiota were also generally lower than that of the UBCs, except for YIDC, as indicated by the Shannon, Simpson, and J indices (**Supplementary Figures 2A–H**). However, most of the differences were not statistically significant, except that YADC, BSC, and QSC gut microbiota had significantly higher microbial richness, diversity, and evenness.

**Figure 2:**
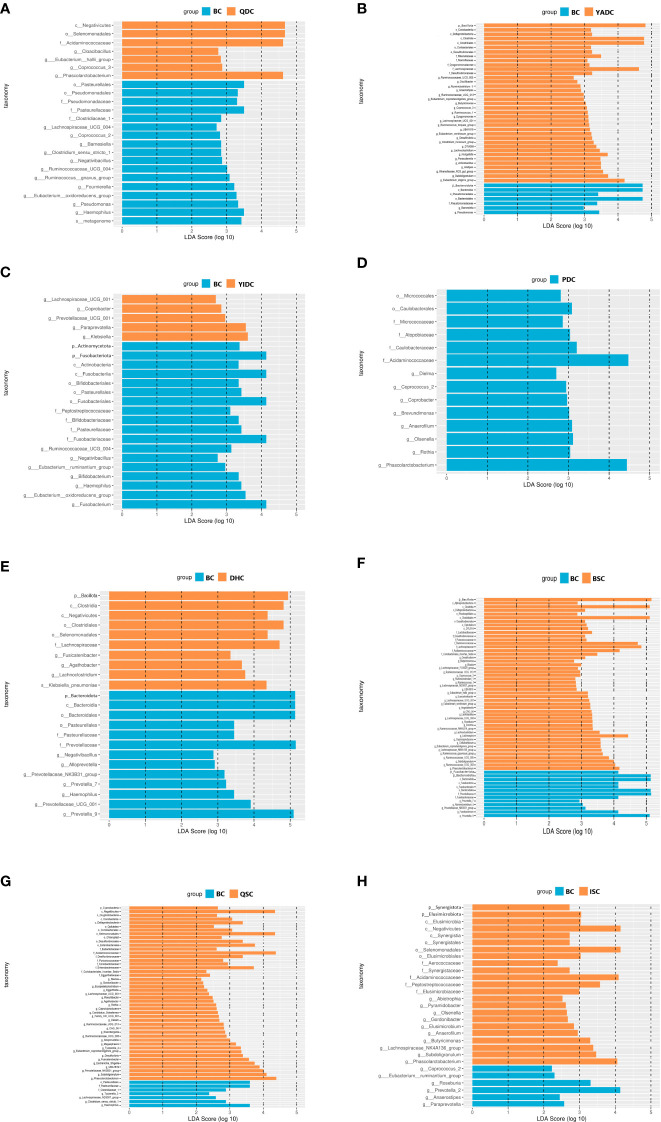
The LEfSe analysis of BC gut microbiota compared with UBCs. **(A)** Linear discriminant analysis (LDA) and effect size analysis (LEfSe) of BC and QDC gut microbiota. **(B)** LEfSe of BC and YADC gut microbiota. **(C)** LEfSe of BC and YIDC gut microbiota. **(D)** LEfSe of BC and PDC gut microbiota. **(E)** LEfSe of BC and DHC gut microbiota **(F)** LEfSe of BC and BSC gut microbiota. **(G)** LEfSe of BC and QSC gut microbiota **(H)** LEfSe of BC and ISC gut microbiota. LEfSe analysis is the distribution circle diagram of linear discriminant analysis of the linear discriminant analysis value, which shows LDA. If the score that is greater than the set value, the statistically different items can be biomarkers. The length of the histogram indicates the influence of the species with a significant indigenous difference. (BC, balanced constitution, *n* = 18; QDC, qi-deficiency constitution, *n* = 16; YADC, yang-deficiency constitution, *n* = 13; YIDC, yin-deficiency constitution, *n* = 11; PDC, phlegm–dampness constitution, *n* = 2; dampness–heat constitution, DHC, *n* = 11; BSC, blood stasis constitution, *n* = 5; QSC, qi-stagnation constitution, *n* = 5; ISC, inherited special constitution, *n* = 7).

To find the specific characterized gut microbiota of the BC population, we did linear discriminant analysis (LDA) and effect size analysis (LEfSe) to find out the taxonomic levels of the different gut microbiota of BC individuals compared with those of each of the UBCs. When compared with the QDC group, 11 genera of gut microbiota were in higher levels of abundance than in the BC group, including *Lachnospiraceae* UCG-004*, Ruminococcaceae* UCG-004, and the *Eubacterium oxidoreducens* group, whereas four genera of gut microbiota were in lower levels, including *Cloacibacillus*, the *Eubacterium hallii* group, and *Phascolarctobacterium* (**Figure 2A**). Two genera of gut microbiota were in higher levels in the BC group than in the YADC group, namely *Pseudomonas* and *Barnesiella*, whereas 25 genera of gut microbiota were in lower levels, including *Hungatella*, *Lachnoclostridium*, and *Ruminococcaceae* UCG-013 (**Figure 2B**). Seven genera of gut microbiota were in higher levels in the BC group than in the YIDC group, including *Negativibacillus*, *Bifidobacterium*, and the *Eubacterium oxidoreducens* group, whereas five genera of gut microbiota were in lower levels, including *Paraprevotella, Coprobacter*, and *Prevotellaceae* UCG-001 (**Figure 2C**). Eight genera of gut microbiota were in lower levels in the BC group than in the PDC group, including *Phascolarctobacterium*, *Olsenella*, *and Coprobacter* (**Figure 2D**). When compared with DHC group, seven genera of gut microbiota were in higher levels in the BC group, including *Prevotella*, *Alloprevotella*, and *Prevotellaceae* UCG-001, whereas three genera of gut microbiota were in lower levels, including *Fusicatenibacter*, *Agathobacter*, and *Lachnoclostridium* (**Figure 2E**). Five genera of gut microbiota were in higher levels in the BC group than in the BSC group, including the *Prevotellaceae* NK3B31 group*, Fusobacterium*, and *Prevotella* 9, whereas 32 genera of gut microbiota were in lower levels, including *Ruminococcaceae* UCG-005, *Ruminococcaceae* UCG-002, and *Lachnospira* (**Figure 2F**). Four genera of gut microbiota were in higher levels in the BC group than in the QSC group, namely *Tyzzerella* 3, the *Lachnospiraceae* ND3007 group, *Clostridium* sensu stricto 1, and *Haemophilus*, whereas 27 genera of gut microbiota were in lower levels, including *Ruminococcaceae* UCG-013, the *Prevotellaceae* NK3B31 group, and *Phascolarctobacterium* (**Figure 2G**). Six genera of gut microbiota were in higher levels in the BC group than in the ISC group, including *Prevotella* 2, *Roseburia*, and the *Eubacterium ruminantium* group, whereas 10 genera of gut microbiota were in lower levels, including *Phascolarctobacterium*, *Butyricimonas*, and the *Lachnospiraceae* NK4A136 group (**Figure 2H**).

### 3.3 FMT donor screening of BC and UBC volunteers

Since people who have a BC are supposed to be the healthiest, we wondered if there were more qualified FMT donors among the BC population. According to previous clinical experience, FMT donor screening was quite costly in terms of time, labor, and money, primarily due to its low success rate; for example, among 100 volunteers that go on to the medical examination step, only one or two will finally be free of any abnormal health conditions and identified as qualified FMT donors (Dubois et al., 2021). Therefore, we recruited BC and UBC volunteers for medical examination following the standard procedure of the updated specialist consensus on FMT donor selection. Among 60 BC volunteers (i.e., 26% of the total volunteers who had a single BC), 20 (33%) became qualified FMT donors, while among the 21 UBC volunteers (i.e., 33% of the total volunteers with single UBC), only four (19%) became qualified FMT donors (**Table 4**). This result shows that it is indeed easier to obtain qualified FMT donors from the BC population than the UBC population.

**Table 4:** The success rate of FMT donor screening among college students with simple single BC and UBC TCM constitutions.

	Total		Male		Female	
**Samples, n**	81		51		30	
**Age, year**	20±2		20±2		20±2	
**BMI, kg/m^2^**	21.02±4.37		21.76		20.32	
**BC n, (%)**	20/60	33.3%	15/40	21.5%	5/20	25.0%
**QDC n, (%)**	1/6	16.7%	1/5	20.0%	0/1	0.0%
**YADC n, (%)**	1/5	20.0%	1/4	25.0%	0/1	0.0%
**YIDC n, (%)**	1/5	20.0%	1/4	25.0%	0/1	0.0%
**PDC n, (%)**	ND	ND	ND	ND	ND	ND
**DHC n, (%)**	1/5	20.0%	1/4	25.0%	0/1	0.0%
**BSC n, (%)**	ND	ND	ND	ND	ND	ND
**QSC n, (%)**	ND	ND	ND	ND	ND	ND
**ISC n, (%)**	ND	ND	ND	ND	ND	ND

BMI, body mass index; BC, balanced constitution; QDC, qi-deficiency constitution; YADC, yang-deficiency constitution; YIDC, yin-deficiency constitution; PDC, phlegm-dampness constitution; DHC, dampness-heat constitution; BSC, blood stasis constitution; QSC, qi stagnation constitution; ISC, inherited special constitution.

### 3.4 Effects of FMT from different TCM constitution donors on mice with HFD-induced obesity

**Figure 3:**
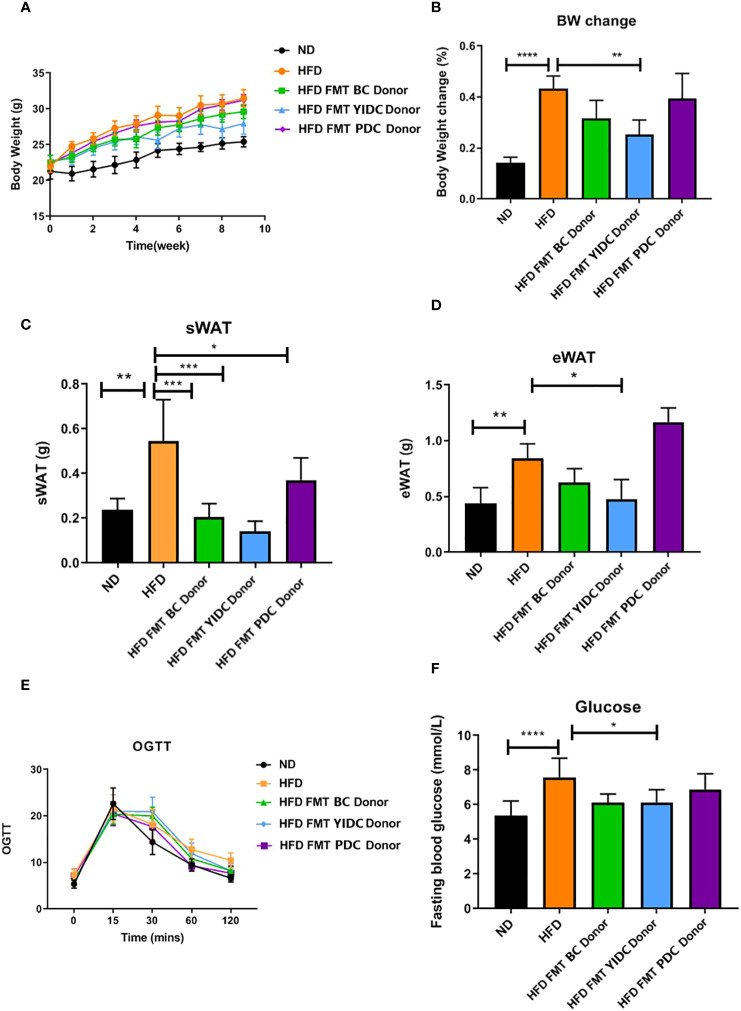
The effects of FMT from different constitution donors on mice with HFD-induced obesity. **(A)** Body weight gain curve. **(B)** Body weight change. **(C)** Subcutaneous fat accumulation. **(D)** Epididymis adipose accumulation. **(E)** OGTT curve. **(F)** Fasting glucose level. (BC donor, BMI = 21 kg/m^2^; YIDC donor, BMI = 19 kg/m^2^; PDC donor, BMI = 23 kg/m^2^.). *p < 0.05; **p < 0.01; and ***p < 0.001, ****p < 0.0001.

To further check if the TCM constitution can provide a useful reference for FMT effects, we compared the FMT effects of the qualified BC and UBC donors using mice with obesity induced by a high-fat diet (HFD), because the majority of different gut microbiota between BC and UBCs were closely associated with obesity and metabolic health, and the BMI was used for measuring obesity. A BC donor (BMI = 21 kg/m^2^), a YIDC donor (BMI = 19 kg/m^2^), and a PDC donor (BMI = 23 kg/m^2^) were chosen for the test, because according to the TCM constitution theory, people with the YIDC constitution are more likely to be thin, and people with the PDC constitution are more likely to have obesity (Zhu et al., 2010; Li et al., 2017; Sun et al., 2018). We observed different FMT outcomes in HFD-fed mice receiving crude fecal microbiota from the BC, PDC, and YIDC donors. Interestingly, HFD-fed mice receiving BC and YIDC fecal microbiota gained less weight than those receiving control and PDC microbiota (**Figures 3A, B**), and those mice also accumulated less subcutaneous fat and epididymis adipose (**Figures 3C, D**). Moreover, BC and YIDC fecal microbiota alleviated the glucose intolerance of HFD-fed mice, as indicated by the OGTT curve and fasting glucose level values (**Figures 3E, F**). These results indicated that TCM constitutions have potential value as references in FMT practice. Meanwhile, the comparisons of the gut microbiota of people with different TCM constitutions and mice in the different treatment groups are presented in **Supplementary Figure 4**.

## 4 Discussion

To explore the characteristics of healthy gut microbiota, our study used the TCM body constitution theory to distinguish the healthiest people among the generally healthy population. A total of 1,218 physically and mentally healthy college students were divided into nine groups, which comprised a group with participants who had a BC, and eight groups of participants who had each of the eight UBCs. The general profiles of the gut microbiota of participants in the BC group and those in each of the eight UBC groups (including in terms of microbial richness, diversity, and composition) were compared. This was a previously unreported idea to explore a healthy gut microbiota and the results provided a new reference for a healthy intestinal ecosystem.

To differentiate this study from previous studies that have tried to describe the gut microbiota profiles of BC and UBC people, our study recruited college students instead of random social volunteers. It is well known that apart from genetic factors, the gut microbiota is also greatly affected by diet, health status, age, and lifestyle. The choice of college students as research participants can eliminate as many interference factors as possible, making TCM constitution a single variable. First, the age of the college students was within a very narrow range (from 18 years to 22 years). Second, the young students were mostly in good health, excluding the interference of various diseases and disease tendencies. Third, the college students led a relatively healthy lifestyle and consumed a relatively healthy diet on campus, eliminating the interference of unhealthy lifestyle tendencies, such as overeating, staying up late, smoking, and drinking. In all, the characteristics of the healthy gut microbiota were expected to be more accurately described through carefully choosing participants and the precise distinguishing of the healthiest people using the TCM constitution theory.

There is a natural connection between TCM constitutions and gut microbiota. There are three basic hypotheses in the TCM constitution theory: (1) people’s TCM constitution changes as they progress through life; (2) people’s appearance and mental spirits can be shaped by their TCM constitution; and (3) people’s TCM constitution is determined by both environmental and genetic factors (Sun et al., 2018). Coincidentally, the latest research on gut microbiota also found that people’s gut microbiota dynamically changed with different developmental periods from birth to aging (O'Toole and Jeffery, 2015; Tanaka and Nakayama, 2017). It was also found that people’s emotion, cognition, and other central nervous system functions were greatly influence by their gut microbiota through a mechanism called the gut–brain axis (Mou et al., 2022). Moreover, people’s gut microbiota is determined by both the host’s genetics and the environmental factors (Goodrich et al., 2014; Rothschild et al., 2018). In addition, there are three key scientific issues related to the TCM constitution theory: (1) how to distinguish a person’s constitutions; (2) how to find the mechanisms that a specific constitution caused specific diseases; and (3) how to regulate a person’s constitution from the unbalanced status to the balanced status. All these issues are inherent to the most important questions in the field of gut microbiota: if people’s gut microbiota, which share similarities but also have big individual differences, can be reduced to several patterns, what is the causal relationship between gut microbiota and specific diseases, and how can gut microbiota be regulated from dysbiosis to normal?

In spite of the obvious connection between TCM constitutions and gut microbiota, there has not been much progress in identifying their specific relationship. One reason for this backwardness may be the lack of study participants in similar conditions and with a single simple TCM constitution. In our study, college students of a similar health status were recruited as study participants and stool specimens were collected only from participants with a strictly simple single constitution. The gut microbiota profiles of each simple single TCM constitution were analyzed. This study, on the one hand, has made a scientific interpretation of TCM constitution theory from the view of modern medicine, and has also provided a scientific reason for using the TCM constitution as a health standard in modern medicine.

Our results indicated there were apparent differences in the profiles of gut microbiota between BC and UBC people. Interestingly, microbial richness, diversity, and evenness were not necessarily higher in BC people, who are believed to be the healthiest according to the TCM theory. In fact, higher microbial richness, diversity, and evenness did not necessarily mean healthier in quite a lot of meta-analyses of patients and their control participants (Jiao et al., 2018; Gong et al., 2021; Illescas et al., 2021; Li et al., 2022). Notably, the components of gut microbiota explained participant health status much better than the richness, diversity, and evenness.

More interestingly, our gut microbiota component analysis of the three simple single TCM constitutions, namely YIDC, YADC, and DHC, found meaningful correlations of gut microbiota and the participant**’**s physique. According to the TCM constitution theory, people who have the YIDC constitution are generally emaciated (Qi, 2005; Sun et al., 2018). Our research found that the relative abundances of skinny-related *Coprobacter* and *Prevotellaceae* UCG-001 were significantly increased (Song et al., 2019; Jie et al., 2021), whereas the relative abundances of obesity-related *Negativibacillus* and the *Eubacterium oxidoreducens* group were reduced in YIDC people when compared with BC people (Zhang et al., 2020; Atzeni et al., 2022).

People who have the YADC or DHC constitution are generally more obese according to the TCM constitution theory. Our results showed that YADC people had higher levels of *Ruminococcaceae* UCG-005, *Ruminococcaceae* UCG-010*, Ruminococcaceae* UCG-013*, Lachnospiraceae* UCG-001, *Lachnoclostridium*, *Parasutterella*, and *Hungatella* than BC people. Among them, *Ruminococcaceae* and *Lachnospiraceae* UCG-001 are involved in the metabolism of various carbohydrates and short-chain fatty acids (SCFAs) that regulate energy metabolism, and their overgrowth can lead to obesity (Walker et al., 2011; Zhang et al., 2017; Liu et al., 2019; Cao et al., 2020; Qin et al., 2022; Shen et al., 2022; Crost et al., 2023). The abundance of *Parasutterella* has been reported to be positively correlated with BMI, type 2 diabetes, and the intake of carbohydrates, and its high abundance has been reported to activate the human fatty acid synthesis pathway, thus leading to weight gain (Henneke et al., 2022). *Lachnoclostridium* and *Hungatella* are trimethylamine-producing bacteria, which have been reported to promote the occurrence and development of atherosclerosis (Genoni et al., 2020; Cai et al., 2022).

As for DHC people, the abundances of *Fusicatenibacter*, *Agathobacter*, and *Lachnoclostridium* were higher, whereas the abundances of *Alloprevotella* and *Prevotellaceae* UCG-001 were lower than in BC people. *Fusicatenibacter* and *Agathobacter* were positively correlated with obesity in children (Vazquez-Moreno et al., 2021; Jiang et al., 2022), whereas *Lachnoclostridium* has been reported to increase significantly in atherosclerosis patients (Cai et al., 2022). *Alloprevotella* has been reported to alleviate weight gain caused by a high-fat diet, and *Prevotellaceae* UCG-001 was found to promote the weight loss in ob/ob mice (Kong et al., 2019; Song et al., 2019).

Most interestingly, our FMT experiment showed that HFD-fed mice receiving the fecal microbiota of the skinny YIDC donor gained less weight and accumulated less subcutaneous and epididymis fat than control mice, whereas mice with obesity receiving the fecal microbiota of a slightly obese PDC donor gained more weight and accumulated more fat. These results provided further proof for meaningful correlations between gut microbiota and the TCM constitution theory, especially in the aspect of physique.

There are many challenges in FMT donor screening. In general, donor screening procedures will go through offline or online questionnaires, face-to-face interviews, and laboratory examination of blood, urine, and stool. The biggest challenge is the low success rate, as the previous experience has shown that qualified donors who pass the laboratory examination constitute no more than 2% of the volunteers who complete the questionnaire. The manpower, time, and economic costs required during the whole process were very high. Our FMT donor screening achieved much higher success rates, that is, 33% from the BC volunteer population, and 19% from the UBC volunteer population. These high success rates were primarily because the study volunteers were young healthy college students. However, the results also showed that it was much easier to obtain qualified FMT donors from the BC population, and a TCM constitution questionnaire may be valuable to improve the success rate, which in turn can decrease manpower, time, and the economic costs of FMT donor screening.

This study had several limitations: First, the sample size of each simple single TCM constitution was limited, especially for several of the UBCs, including PDC, BSC, QSC, and ISC. The number of these samples was quite small due to a very low proportion of the whole population having these types of UBCs, resulting in a slight shortage of data for gut microbiota analysis and donor screening. Second, the TCM constitution questionnaire itself has limitations, since answers to the questions may not be consistently objective among individuals, since participants’ self-confidence may affect their feelings regarding their body conditions, thus confident and positive people are more likely to be identified as BC. Third, diet is an important factor affecting gut microbiota, and, although the dietary questionnaire of each student was collected along with the TCM questionnaire, the majority of the students reported having a generally balanced nutrition, including enough diversified staple foods, vegetables, and meat (data not shown), the volunteers were not required to control their diet before sampling, and, therefore, we could not rule out interference related to an occasionally indulgent diet. Fourth, although our FMT experiments on mice had interesting results indicating that the fecal microbiota of different TCM constitutions conferred different traits; the mechanism of this was not yet clear and rodent results often cannot be translated to humans, thus, further clinical trials will be needed to verify the value of TCM constitution in the matching of donor–receptor in FMT, and this mechanism needs further study.

## 5 Conclusion

The hypotheses of the traditional Chinese medicine (TCM) constitution theory coincidentally align with the key research findings on gut microbiota. Our study made efforts to eliminate the various confounding factors related to the gut microbiota to explore the association between TCM constitution and gut microbiota. The results revealed significant differences in gut microbiota among the nine TCM constitutions. Compared with the unbalanced constitutions (UBCs), the balanced constitution (BC), which is considered the healthiest according to TCM constitution theory, showed a predominance of beneficial bacterial species in the gut microbiota. Several UBCs, such as the yin-deficiency, yang-deficiency, and phlegm-dampness constitutions, had gut microbiota characteristics that could even partially explain participants’ corresponding body fat and their being overweight or underweight. This further indicates that the TCM constitution theory may obtain scientific explanations from the perspective of the gut microbiota. By incorporating the TCM constitution questionnaire into the screening of FMT donors, a higher success rate can be achieved from the BC population. FMT experiments on mice demonstrated that donors with different TCM constitutions conferred different effectiveness. These findings suggest that the TCM constitution theory could provide valuable references for the screening of FMT donors, and BC donors may potentially enhance the therapeutic effects of FMT. However, these hypotheses require further validation through more samples and clinical experiments.

## Data availability statement

The data presented in the study are deposited in the SAR NCBI repository, accession number PRJNA876832.

## Ethics statement

The studies involving humans were approved by the Fifth Affiliated Hospital of Guangzhou Medical University (ref. KY02-2022-08-02). The studies were conducted in accordance with the local legislation and institutional requirements. The participants provided their written informed consent to participate in this study. The animal study was approved by the Fifth Affiliated Hospital of Guangzhou Medical University (ref. KY01-2020-03-11). The study was conducted in accordance with the local legislation and institutional requirements.

## Author contributions

QH: Writing – review & editing. LY: Investigation, Writing – original draft. GC: Investigation, Writing – original draft. YH: Software, Writing – original draft. SZ: Data curation, Writing – original draft. ZY: Investigation, Writing – original draft. JY: Methodology, Writing – original draft. CG: Data curation, Writing – original draft. JL: Methodology, Writing – original draft. LL: Data curation, Writing – original draft. JW: Writing – review & editing. TL: Supervision, Writing – original draft.

## Supplementary material

Supplementary Figure 1 The PCoA of BC gut microbiota compared with UBCs. The principal component analysis (PCoA) scatter plot of BC and each UBC gut microbiota. Each point in the figure is a sample, and different colors represent different experimental groups. The closer the points are, the more similar the samples are. (BC, balanced constitution, *n* = 18; QDC, qi-deficiency constitution, *n* = 16; YADC, yang-deficiency constitution, *n* = 13; YIDC, yin-deficiency constitution, *n* = 11; PDC, phlegm–dampness constitution, *n* = 2; dampness–heat constitution, DHC, *n* = 11; BSC, blood stasis constitution, *n* = 5; QSC, qi-stagnation constitution, *n* = 5; ISC, inherited special constitution, *n* = 7.)

Supplementary Figure 2 The β-diversity of BC gut microbiota compared with UBCs. **(A)** The α-diversity of BC and QDC gut microbiota. **(B)** The α-diversity of BC and YADC gut microbiota. **(C)** The α-diversity of BC and YIDC gut microbiota. **(D)** The α-diversity of BC and PDC gut microbiota. **(E)** The α-diversity of BC and DHC gut microbiota **(F)** The α-diversity of BC and BSC gut microbiota. **(G)** The α-diversity of BC and QSC gut microbiota **(H)** The α-diversity of BC and ISC gut microbiota. The α-diversity includes the bacterial richness observation species, and the Chao1 and ACE index values, and the bacterial diversity Shannon and Simpson indices. (BC, balanced constitution, *n* = 18; QDC, qi-deficiency constitution, *n* = 16; YADC, yang-deficiency constitution, *n* = 13; YIDC, yin-deficiency constitution, *n* = 11; PDC, phlegm–dampness constitution, *n* = 2; dampness–heat constitution, DHC, *n* = 11; BSC, blood stasis constitution, *n* = 5; QSC, qi-stagnation constitution, *n* = 5; ISC, inherited special constitution, *n* = 7.)

Supplementary Figure 3 The differential phylotypes in abundance between BC and UBCs. **(A)** Differential phylotypes in abundance between BC and QDC. **(B)** Differential phylotypes in the abundance between BC and YADC. **(C)** Differential phylotypes in the abundance between BC and YIDC. **(D)** Differential phylotypes in the abundance between BC and PDC. **(E)** Differential phylotypes in the abundance between BC and DHC. **(F)** Differential phylotypes in the abundance between BC and BSC. **(G)** Differential phylotypes in the abundance between BC and QSC **(H)** Differential phylotypes in the abundance between BC and ISC.

Supplementary Figure 4 Comparison of gut microbiota of human with different TCM constitution and mice in different treatment groups. **(A)** PCoA scatter plot of gut microbiota of BC human, HFD mice, and HFD mice receiving FMT from BC donor. **(B)** PCoA scatter plot of gut microbiota of YIDC human, HFD mice, and HFD mice receiving FMT from YIDC donor. **(C)** PCoA scatter plot of gut microbiota of PDC human, HFD mice, and HFD mice receiving FMT from PDC donor. **(D)** PCoA scatter plot of gut microbiota of HFD mice and HFD mice reserving its own fecal microbiota. Each point in the figure is a sample, and different colors represent different experimental groups. The closer the points are, the more similar the samples are. **(E)** Bar chart of gut microbiota relative abundance at order level.
