# Supplementary material for: Comparison of the gut microbiota of college students with the nine balanced and unbalanced traditional Chinese medicine constitutions and its potential application in fecal microbiota transplantation
Source: Front Microbiomes. 2023 Dec 20;2:1292273. doi: 10.3389/frmbi.2023.1292273 (PMC12993572; doi:10.3389/frmbi.2023.1292273)
Supplement: Supplementary file 1 [file Table_1.docx]

#### Supplemental Table 1 Identification of TCM Constitution

| **Types of TCM constitution** | **Conditions** | **Results** |
| --- | --- | --- |
| **Balanced constitution (BC)** | Conversion score ≥60, with the conversion score of all the other 8 UBC <30 | BC |
|  |  |  |
|  | Conversion score ≥60, with the conversion score of all the other 8 UBC≥30 and <40 | Inclined BC |
|  |  |  |
|  | Conversion score <60 | UBC |
| **Un-balanced constitution (UBC)** | Conversion score ≥40 | UBC |
|  | Conversion score ≥30 and <40 | Inclined UBC |
|  | Conversion score<30 | Denied UBC |
| Conversion score = [(raw score - number of items) / (number of items ×4)] ×100 | | |
| Raw score = total score of each item | | |
| Un-balanced constitutions (UBCs): the qi-deficiency constitution (QDC), the yang-deficiency constitution (YADC), the yin-deficiency constitution (YIDC), the phlegm dampness constitution (PDC), the dampness heat constitution (DHC), the blood stasis constitution (BSC), the qi stagnation constitution (QSC) and the inherited special constitution (ISC). | | |
