# Supplementary figures and images for: Comparison of the gut microbiota of college students with the nine balanced and unbalanced traditional Chinese medicine constitutions and its potential application in fecal microbiota transplantation
Source: Front Microbiomes. 2023 Dec 20;2:1292273. doi: 10.3389/frmbi.2023.1292273 (PMC12993572; doi:10.3389/frmbi.2023.1292273)

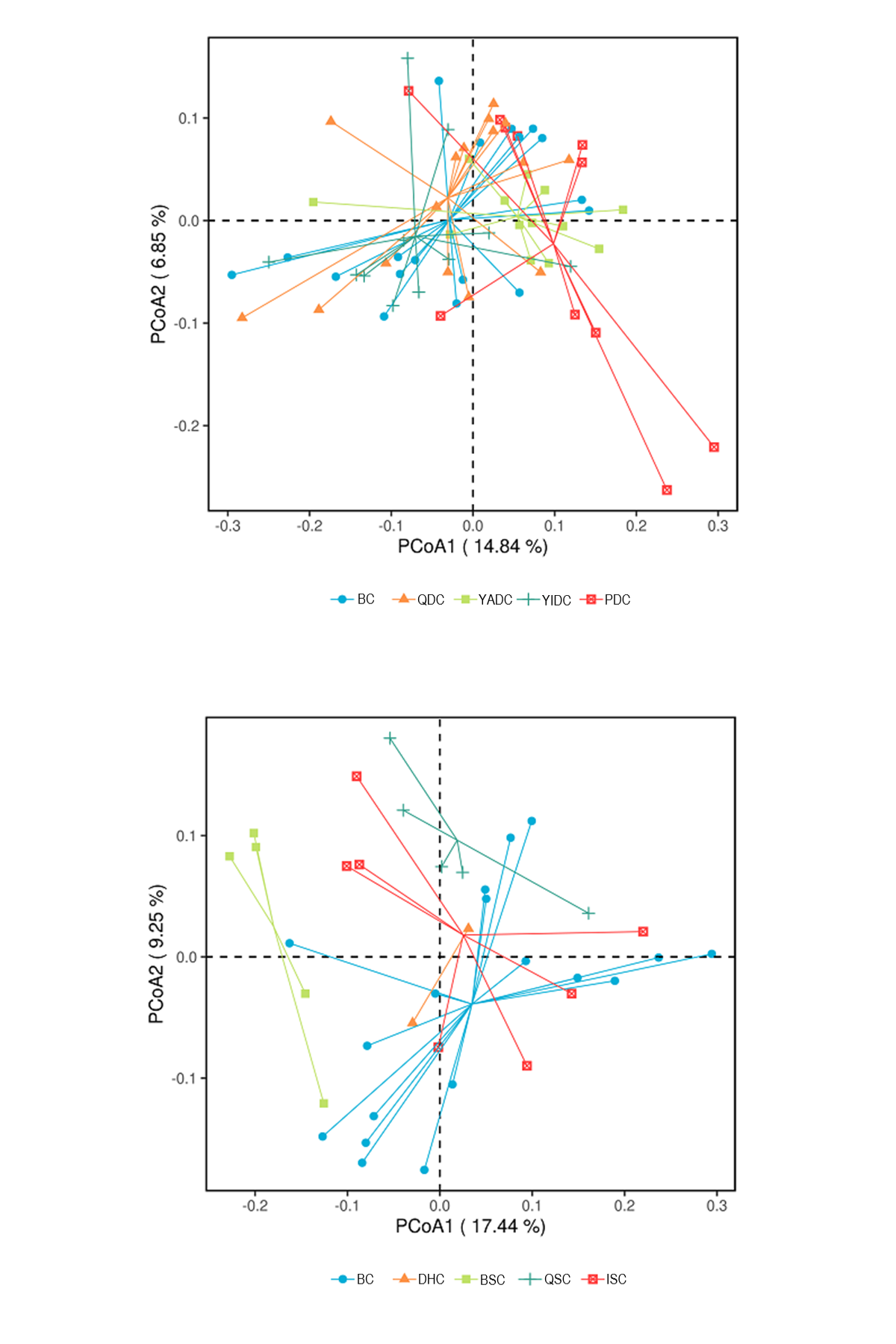

Supplement: Supplementary Figure 1 — The PCoA of BC gut microbiota compared with UBCs. The principal component analysis (PCoA) scatter plot of BC and each UBC gut microbiota. Each point in the figure is a sample, and different colors represent different experimental groups. The closer the points are, the more similar the samples are. (BC, balanced constitution, n = 18; QDC, qi-deficiency constitution, n = 16; YADC, yang-deficiency constitution, n = 13; YIDC, yin-deficiency constitution, n = 11; PDC, phlegm–dampness constitution, n = 2; dampness–heat constitution, DHC, n = 11; BSC, blood stasis constitution, n = 5; QSC, qi-stagnation constitution, n = 5; ISC, inherited special constitution, n = 7.) [file Image_1.tif]

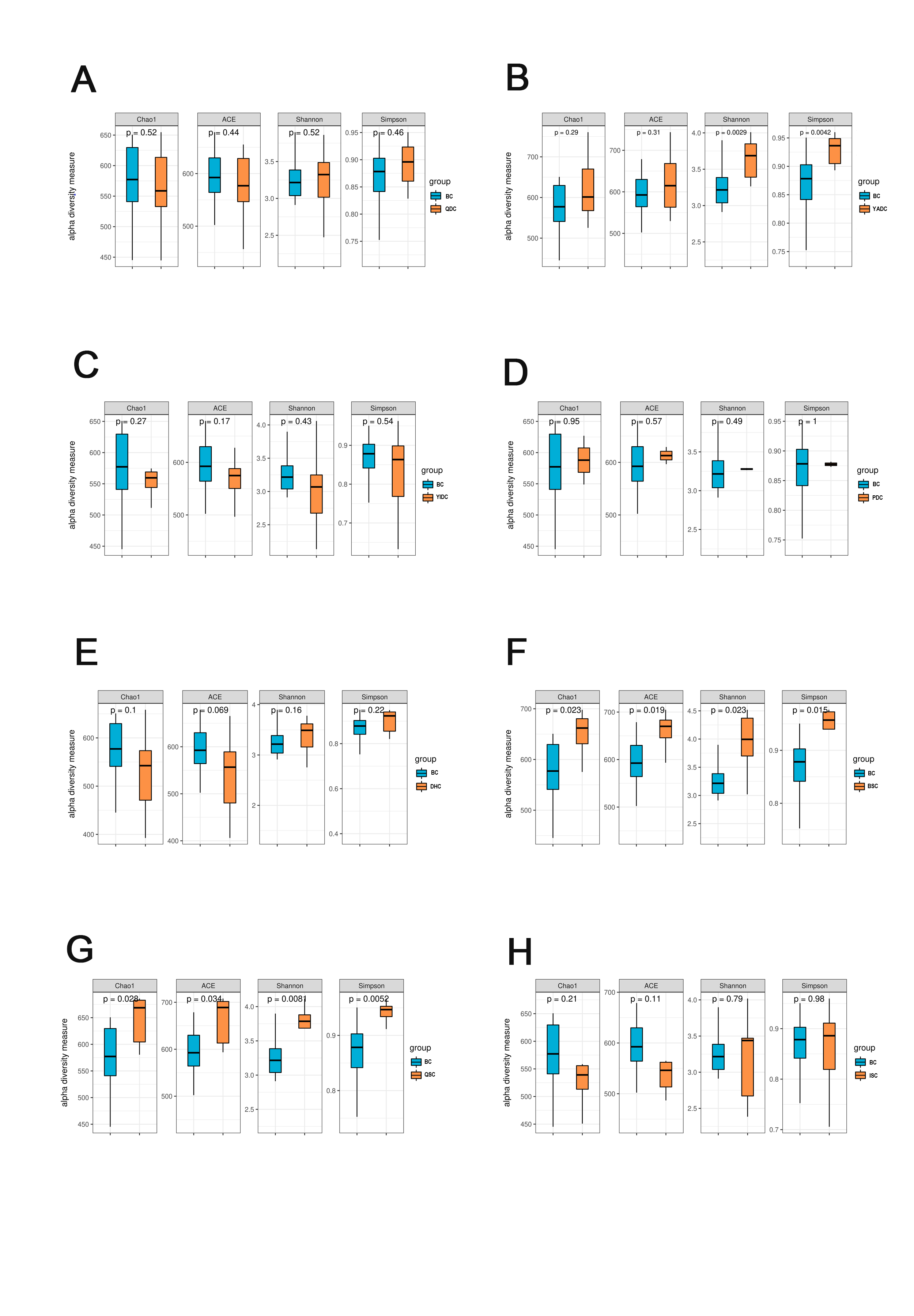

Supplement: Supplementary Figure 2 — The β-diversity of BC gut microbiota compared with UBCs. (A) The α-diversity of BC and QDC gut microbiota. (B) The α-diversity of BC and YADC gut microbiota. (C) The α-diversity of BC and YIDC gut microbiota. (D) The α-diversity of BC and PDC gut microbiota. (E) The α-diversity of BC and DHC gut microbiota (F) The α-diversity of BC and BSC gut microbiota. (G) The α-diversity of BC and QSC gut microbiota (H) The α-diversity of BC and ISC gut microbiota. The α-diversity includes the bacterial richness observation species, and the Chao1 and ACE index values, and the bacterial diversity Shannon and Simpson indices. (BC, balanced constitution, n = 18; QDC, qi-deficiency constitution, n = 16; YADC, yang-deficiency constitution, n = 13; YIDC, yin-deficiency constitution, n = 11; PDC, phlegm–dampness constitution, n = 2; dampness–heat constitution, DHC, n = 11; BSC, blood stasis constitution, n = 5; QSC, qi-stagnation constitution, n = 5; ISC, inherited special constitution, n = 7.) [file Image_2.tif]

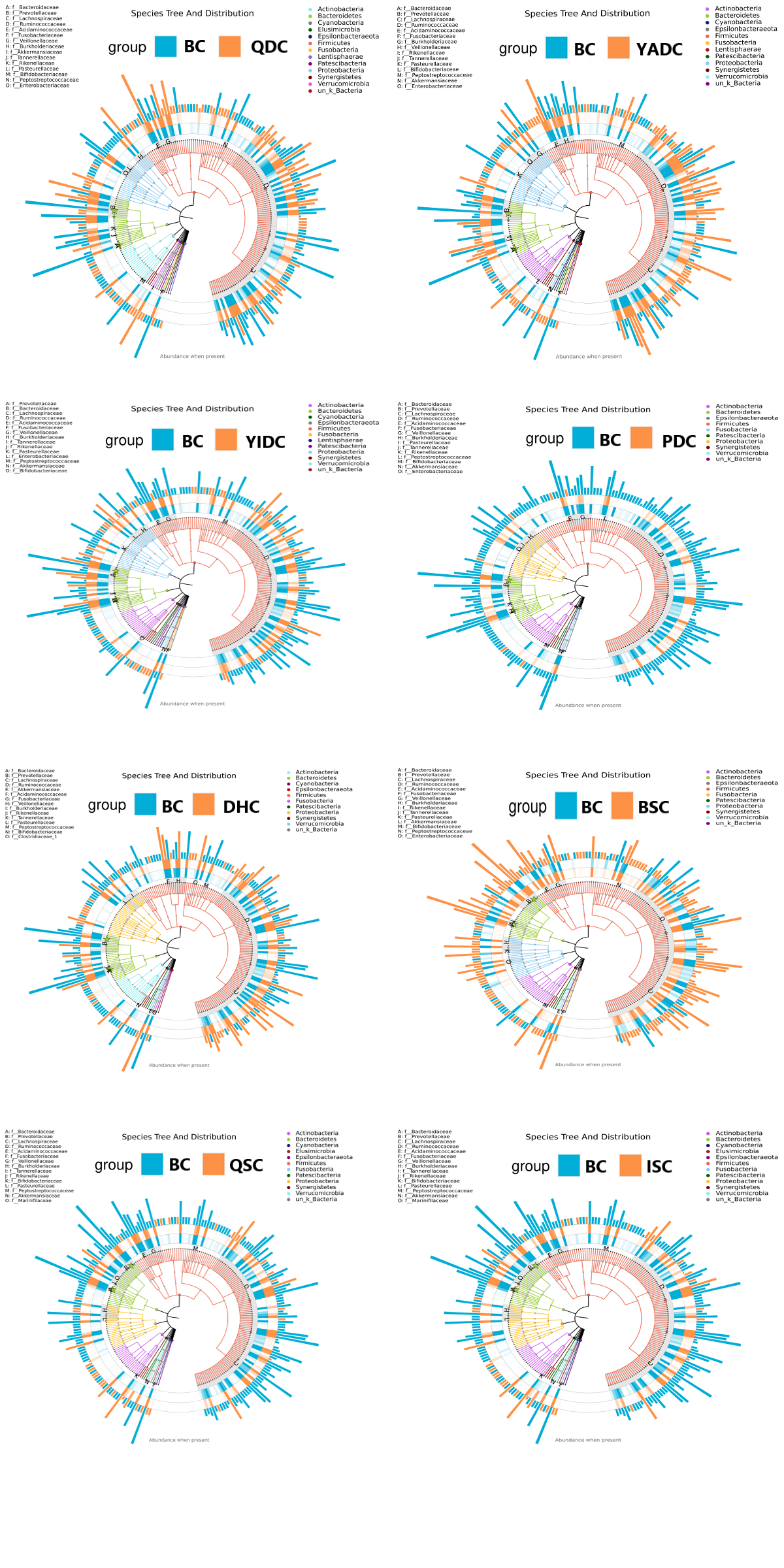

Supplement: Supplementary Figure 3 — The differential phylotypes in abundance between BC and UBCs. (A) Differential phylotypes in abundance between BC and QDC. (B) Differential phylotypes in the abundance between BC and YADC. (C) Differential phylotypes in the abundance between BC and YIDC. (D) Differential phylotypes in the abundance between BC and PDC. (E) Differential phylotypes in the abundance between BC and DHC. (F) Differential phylotypes in the abundance between BC and BSC. (G) Differential phylotypes in the abundance between BC and QSC (H) Differential phylotypes in the abundance between BC and ISC. [file Image_3.tif]

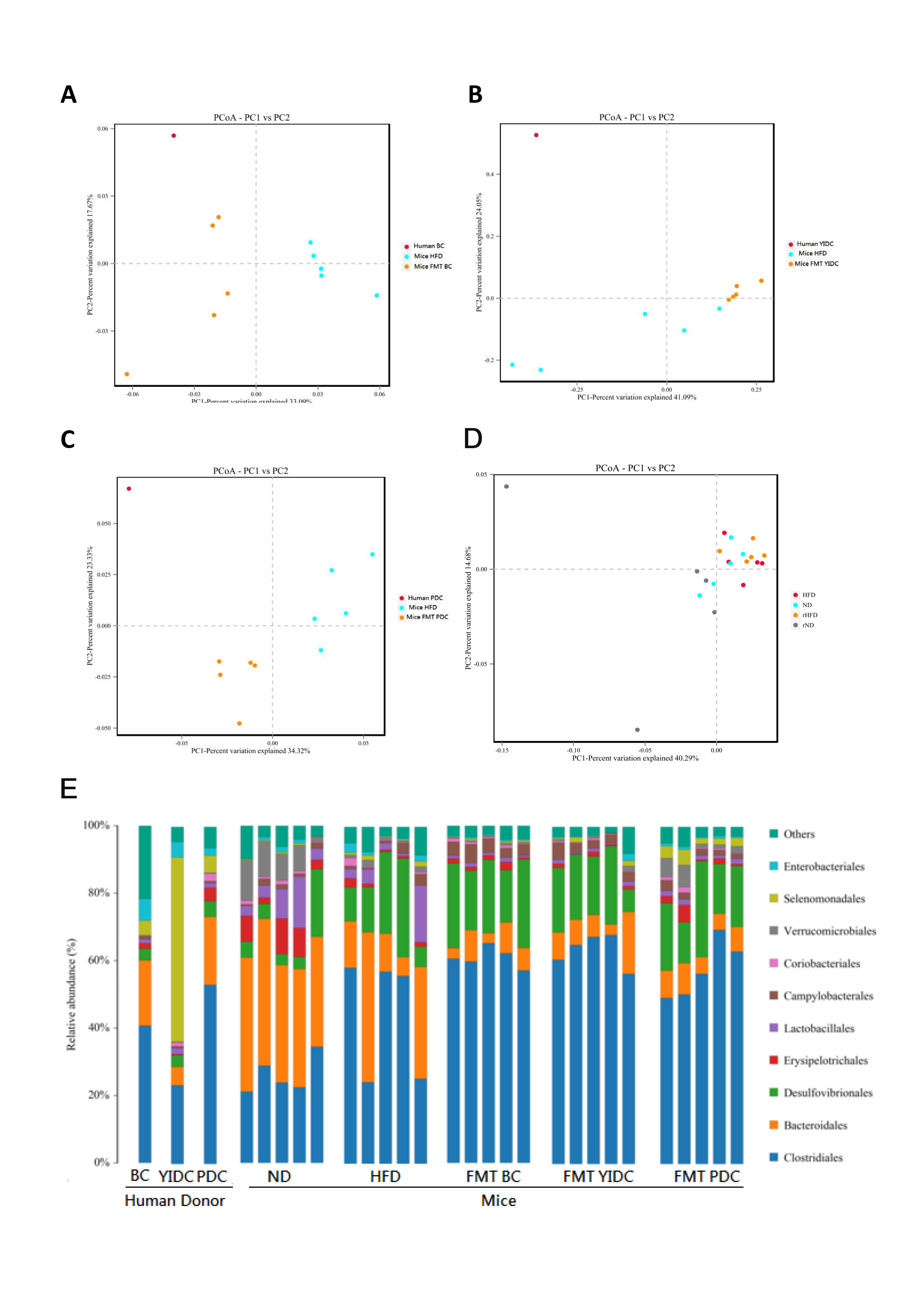

Supplement: Supplementary Figure 4 — Comparison of gut microbiota of human with different TCM constitution and mice in different treatment groups. (A) PCoA scatter plot of gut microbiota of BC human, HFD mice, and HFD mice receiving FMT from BC donor. (B) PCoA scatter plot of gut microbiota of YIDC human, HFD mice, and HFD mice receiving FMT from YIDC donor. (C) PCoA scatter plot of gut microbiota of PDC human, HFD mice, and HFD mice receiving FMT from PDC donor. (D) PCoA scatter plot of gut microbiota of HFD mice and HFD mice reserving its own fecal microbiota. Each point in the figure is a sample, and different colors represent different experimental groups. The closer the points are, the more similar the samples are. (E) Bar chart of gut microbiota relative abundance at order level. [file Image_4.tif]
